# Vallecular Cyst in Neonates: Case Series—A Clinicosurgical Insight

**DOI:** 10.1155/2014/764860

**Published:** 2014-10-28

**Authors:** Shweta Gogia, Sangeet Kumar Agarwal, Alok Agarwal

**Affiliations:** Department of Otolaryngology and Head, Neck Surgery, Sir Ganga Ram Hospital, New Rajinder Nagar, New Delhi 110060, India

## Abstract

The objective of the case series is to understand the clinical and surgical aspects of new minimally invasive technique of coblation in cases of vallecular cysts in neonates. *Method of Study*. Four neonates underwent surgery for vallecular cyst by using Arthrocare ENT coblator system. *Results Obtained*. All the four cases presented in stridor and difficult intubation was also a concern which necessitated a swift, high precision instrument with almost immediate results. Coblation excision includes direct contact with vallecular cyst, improved targeting of the cyst, and preservation of normal tissue. All the four cases had an uneventful postoperative period and smooth recovery and had an early discharge from the hospital. *Conclusions*. Early diagnosis and intervention hold the key for an early recovery and for minimizing nutritional disturbances secondary to poor feeding in cases of neonatal vallecular cysts.

## 1. Introduction

Congenital vallecular cyst is a rare but potentially dangerous cause of stridor in neonates and young infants. Without recognition and proper therapy, the resulting airway obstruction can lead to serious morbidity and mortality [1–9]. When seen in adults, vallecular cysts are asymptomatic or with subtle symptoms such as voice change [4]; in contrast, it can cause stridor and/or respiratory distress in neonates and young infants due to their small airway [1]. It also has potential to excite retching reflex, which may induce gagging and vomiting which may lead to feeding difficulties and failure to thrive [1, 2, 5, 6]. Even though there have been case reports in the literature, we emphasize that the condition needs to be identified by pediatricians and general practitioners and managed appropriately. We here also describe a novel approach in the management of vallecular cyst in a series of 4 cases using coblation. This surgical tool considerably improved the surgical outcome by decreasing the morbidity and total duration of hospital stay. In this case series, an attempt has been made to give insight into the overall prognosis and recovery of the patient after using coblation technology in this particular area of pediatric anatomy.

### 1.1. Case I

**Figure 1:**
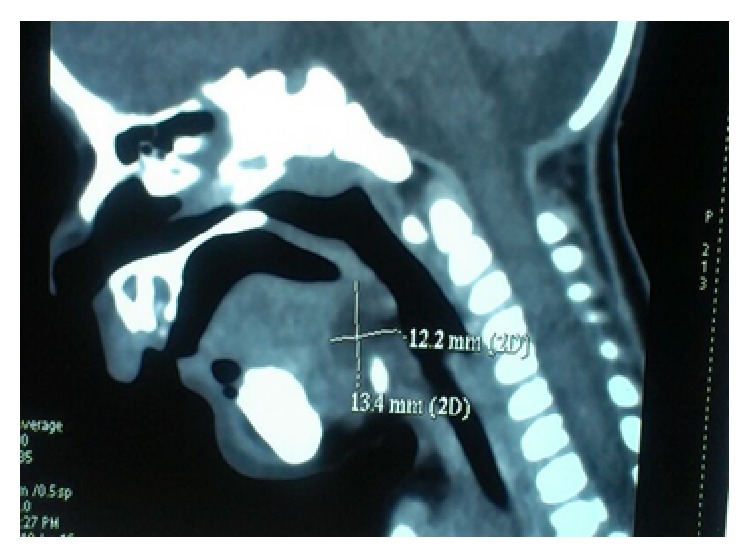
Contrast enhanced computed tomographic scan showing nonenhancing fluid filled lesion located at base of tongue of 12.2 × 13.4 mm.

A 2-month-old term female baby was referred to us for noisy breathing and progressive respiratory distress. Earlier, the child developed respiratory distress with failure to thrive and noisy breathing. On examination, vitals were stable and there were no dysmorphic features. She had marked inspiratory stridor with suprasternal and subcostal recession and tachypnoea. A provisional diagnosis of severe laryngomalacia was made. A flexible fiberoptic bronchoscopy was done which revealed a cystic mass at the base of the tongue which was obstructing the laryngeal inlet and was pushing the epiglottis. Computed tomography scan showed well-defined nonenhancing fluid filled lesion located at base of tongue 12.2 × 13.4 mm in size (Figure 1) causing significant narrowing of laryngeal inlet. A differential diagnosis of thyroglossal cyst or a vallecular cyst was made.

**Figure 2:**
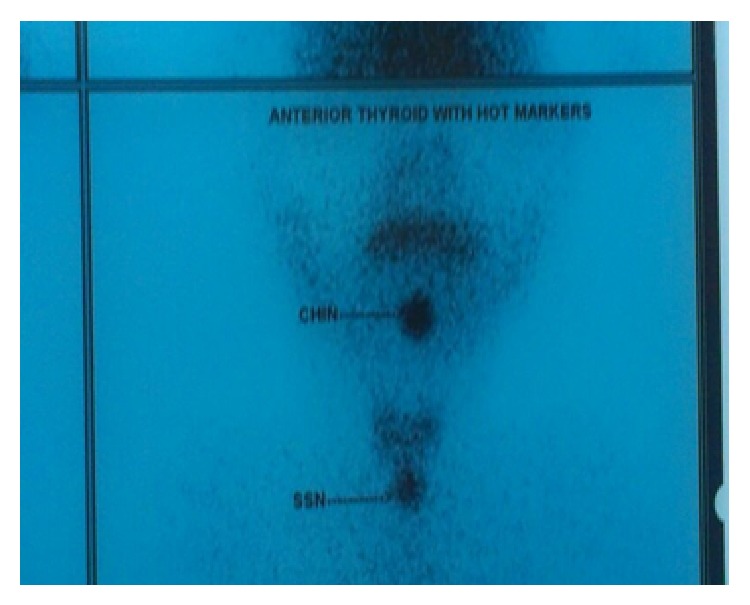
99 m technetium pertechnetate scan. No abnormal tracer uptake was seen in the clinically palpable swelling at the base of the tongue.

99 m technetium (Tc) pertechnetate scan was performed. Both lobes of thyroid gland were normal in size, shape, and placement. No abnormal tracer uptake was seen in the swelling at the base of the tongue (Figure 2).

T3, T4, and TSH levels were also found to be within normal limits.

**Figure 3:**
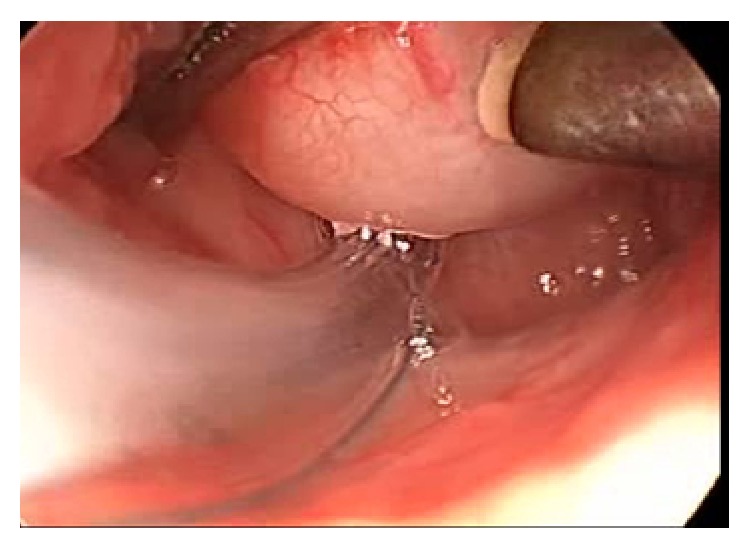
Preoperative endoscopic view of cystic mass of the tongue mass of case I.

**Figure 4:**
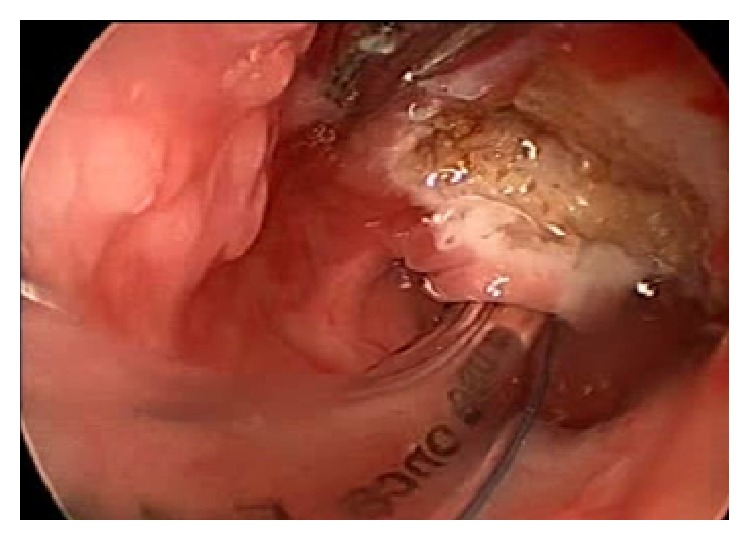
Postoperative endoscopic view of case I.

The child was taken up for coblation assisted ablation of the cystic mass of the tongue mass under endoscopic guidance (Figure 3). An EVac Xtra HP Wand was used with power setting of 7 for ablation and 3 for coagulation. The operating time was about 18 minutes. The blood loss was about 4 mL (Figure 4).

The child had an uneventful recovery with extubation at 24 hours after procedure and thriving well at 3-month follow-up.

### 1.2. Case II

A term 3-month-old male baby was referred to our centre for progressive difficulty in breathing. He was asymptomatic until 1 month of age when he gradually developed stridor and progressive respiratory stress. He was then referred to our centre, where the child again had an apneic spell requiring resuscitation and was subsequently intubated.

**Figure 5:**
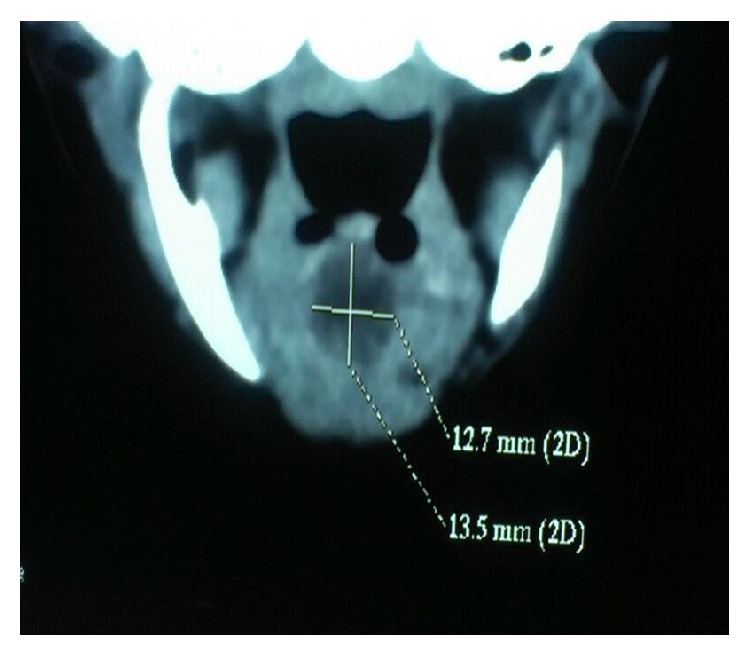
Contrast enhanced computed tomographic scan showing nonenhancing cystic lesion located at the base of the tongue measuring 12.7 mm × 13.5 mm.

Flexible fiberoptic bronchoscopy was done which revealed a large swelling at the base of tongue obscuring the view of laryngeal inlet. Contrast enhanced computed tomographic scan of neck was performed, which showed a well-defined, nonenhancing cystic lesion located at the base of the tongue measuring 12.7 mm × 13.5 mm (Figure 5). The diagnosis of a vallecular cyst was made. 99 m Tc pertechnetate thyroid scan and thyroid profile were done which were found normal. A coblation assisted removal of cystic lesion was done and child was extubated uneventfully 24 hours later. 2-month follow-up showed complete resolution of symptoms and no stridor.

### 1.3. Case III

A 40-day-old term male child was referred to our center for respiratory distress. The child developed noisy breathing about 10 days after birth which continued to worsen. On examination, child was tachypnoeic with mild dehydration. He was having inspiratory stridor and was using accessory respiratory muscles. A flexible fiberoptic bronchoscopy was done which showed a large mass in the base of tongue close to valleculae, thus causing epiglottis to obstruct the laryngeal inlet.

A contrast enhanced computed tomography scan was done which revealed a cystic lesion at base of tongue of 11.7 × 10.3 mm causing significant airway compromise. Thyroid scan revealed that there was no tracer uptake in the region of thyroid gland. No functioning thyroid tissue was appreciated on thyroid scan. A low TSH of 4.7 U/mL was present. The child was started on levothyroxine. The child was taken up for coblation assisted removal of the cyst and was extubated uneventfully 24 hours later.

### 1.4. Case IV

A 4-month-old male was referred with complaints of failure to thrive, noisy breathing, and occasional respiratory distress. The child was a full term normal vaginal delivery; he developed noisy breathing at 15 days of age. The child had frequent episodes of vomiting with features of failure to thrive. The child was treated as a case of laryngobronchomalacia with reflux disease. A contrast enhanced computed tomography scan imaging revealed cystic mass lesion of 10.3 × 8.9 mm in base of tongue compromising the airway due to mass effect on epiglottis.

The child was taken up for removal of cyst using coblation. Endoscopic visualization of the cyst was done. The cyst was ablated using coblation.

## 2. Discussion

Vallecular cyst although rare is a potentially life threatening condition causing sudden airway obstruction by its location [1–5]. 12–45% of cases of laryngomalacia present with synchronous airway abnormality such as laryngeal cyst [3, 9]. Thus, while evaluating neonatal stridor, airway anatomy and differential diagnosis from other causes of stridor are very important in the management of such cases.

Vallecular cysts have been reported in various case studies under differential names [2]. Terms used have included mucus retention cyst, epiglottic cyst, base of the tongue cyst, congenital cyst, and more recently ductal cyst. The term ductal cyst originates from the classification of DeSanto et al. [10] in which they grouped laryngeal cysts according to their location and surface mucosa. Newman classified laryngeal cysts as epithelial, tonsillar, and oncolytic cysts [8].

Vallecular cyst is a unilocular cystic mass of variable size which arises from lingual surface of epiglottis and contains clear and noninfected fluid [2]. Two major theories to explain the pathogenesis of vallecular cyst are that it is a consequence of either ductal obstruction of mucus glands or an embryological malformation [10]. Histologically, the cyst contains respiratory epithelium with mucous glands, with an external lining of squamous epithelium [11].

The infants with vallecular cysts usually present with symptoms during the first few weeks of life [3, 4]. Clinical manifestations consist of various forms of upper airway obstruction such as inspiratory stridor, chest retraction, apnea, cyanosis, and feeding difficulty [1–8].

Infants with vallecular cysts may present with a secondary form of laryngomalacia which can be explained by altered airway dynamics caused by progressively enlarging cyst which cause increased inspiratory negative pressures, contributing to supraglottic prolapse [1].

Flexible laryngoscopy or bronchoscopy is usually performed to diagnose the vallecular cyst [5, 6]. Therefore, special attention must be directed to the area of valleculae and base of tongue while performing flexible bronchoscopy as this may be missed and may lead to misdiagnosis.

In our case series, all cases underwent contrast enhanced computed tomographic scan to see the extent and nature of the mass. A thyroid screening was also performed to rule out the lingual thyroid and thyroglossal cyst and was supplemented by a thyroid scan. In one of the cases, there was total absence of any functioning thyroid tissue which was detected by the thyroid scan.

The smallest child was of 40 days old and inspiratory stridor indicated that nature of lesion was arising from supraglottic and/or glottis region. So, the underlying “ball valve” effect should be kept in mind. However, laryngomalacia should also be excluded [12–14].

Haemangioma, cystic hygroma, teratoma, hamartoma, dermoid cyst, lymphangioma, thyroglossal duct cyst, and thyroid remnant cyst should be considered in the differential diagnosis of vallecular cyst [2–4].

Coblation assisted ablation of the vallecular cyst in our case series was a novel technique for the management of the condition. Coblation is a unique modality that can ablate tissue by generating a field of ionized sodium molecules. During the procedure, conductive saline solution is converted to an ionized plasma layer in the gap between the device tip and the tissue. At the tissue plasma interface, there is generation of adequate energy. This energy breaks the molecular bonds resulting in molecular dissociation. This effect is achieved at temperatures of approximately 40–70°C; so, thermal damage is minimal as compared to radiofrequency. This minimally invasive technology resulted in less tissue trauma, minimal bleeding, and reduced postoperative recovery time, thus effectively resolving the problem of hemorrhage and postoperative decannulation.

## 3. Conclusion

The four cases that we described illustrate how a benign, self-limiting swelling can be potentially life threatening to airway and the superior postoperative outcome secondary to coblation assisted intervention. All the four cases presented in stridor and difficult intubation was also a concern which necessitated a swift, high precision instrument with almost immediate results.

The advantages of coblation excision include direct contact with epiglottic cyst, improved targeting of the cyst and preservation of normal tissue, and lower temperatures to prevent excessive burning, thus improving recovery time and minimizing surgical and postsurgical complications; so, it is a simple, safe, and effective method.
